# Differences in postoperative pain management in patients with Ileitis Crohn and diverticulitis– a matched pair analysis

**DOI:** 10.1007/s00423-026-03989-5

**Published:** 2026-03-06

**Authors:** Regina Pistorius, Anna Widder, Marleen Sabisch, Michael Meir, Imad Maatouk, Christian Markus, Alexander Brack, Patrick Meybohm, Christoph-Thomas Germer, Nicolas Schlegel, Matthias Kelm, Sven Flemming

**Affiliations:** 1 Department of General, Visceral, Transplant, Vascular and Pediatric Surgery, University Hospital Würzburg, Oberduerrbacher Str. 6, Würzburg, 97080 Germany; 2 Department of Internal Medicine, Division of Psychosomatic, Psychotherapy und Psychooncology, University Hospital Würzburg, Würzburg, Germany; 3 Department of Anaesthesiology, Intensive Care, Emergency and Pain Medicine, University Hospital Würzburg, Würzburg, Germany

**Keywords:** Colon resection, Pain, Peridural catheter, Analgesics score

## Abstract

**Purpose:**

Crohn’s disease (CD) belongs to the group of chronic inflammatory bowel diseases (IBD) and is characterized by disease relapse with gastrointestinal symptoms and abdominal pain. Despite continuous development of therapeutical strategies, two thirds of patients suffering from CD need surgical intervention during their lives due to disease progression and complications. It is assumed that CD patients have increased pain experience in the perioperative setting compared to non-IBD patients, but clinical data are rare.

**Methods:**

A retrospective single-centre analysis was performed including patients with Ileitis Crohn who underwent open or minimal-invasive (laparoscopic) ileocecal resection between 2017 and 2021. The cohort was compared to patients who received open or minimal-invasive sigmoid resection due to diverticulitis. A descriptive analysis and comparison of pre-existing conditions (e.g. laboratory values, depression, preoperative (co-)analgesics use), postoperative complications, postoperative pain and pain medication was carried out in both cohorts. A matched-pair analysis was performed to determine disease-related pain levels of patients with CD in comparison to non-IBD patients. Both a previously established analgesics and a validated pain scores were used to compare the two groups.

**Results:**

A total of 310 patients, 182 non-IBD patients and 128 CD patients, were analysed. According to Clavien Dindo classification, there were significantly more postoperative complications in CD patients (> 3a) compared to the non-IBD cohort (14.8% vs. 5.5%; *p* = 0.005). CD patients showed a significantly increased need for metamizole (89.1% vs. 73.1%; *p* < 0.001), and co-analgesics (18% vs. 4.4%; *p* < 0.001) postoperatively. Furthermore, the duration of intake of high-potent opioids was significantly longer compared to non-IBD patients (2.4 vs. 1.2 [days]; *p* = 0.038). These differences in postoperative pain management resulted in equal postoperative pain experience and sensation in patients suffering from Ileitis Crohn and diverticulitis as revealed by NRS scores. Interestingly, a matched-pair analysis between patients with limited CD and non-IBD patients (2x *n* = 27) showed neither significant differences in postoperative pain levels, need for additional analgesics nor the analgesic score.

**Conclusions:**

Postoperative analgesic regimen need to consider the individual patient’s pain score, pain experience and sensation irrespective of the underlying disease leading to colorectal surgery.

## Introduction

Crohn`s disease (CD) as one of the main representatives of inflammatory bowel diseases (IBD) is characterized by chronic, disease-related pain. The dysregulated inflammatory response in CD patients exacerbates visceral pain and may cause hyperalgesia [1]. Despite the successful implementation of medical strategies including biologicals, the rate of patients who suffer from complicated CD and require surgery remains stable over the decades with approximately two thirds of patients with CD that need abdominal surgery [2].

While pre-existing inflammation can impair the perioperative course significantly, surgical interventions are also associated with an increased inflammatory reaction perioperatively caused by various mediators and biochemical processes [3, 4]. Thus, the perioperative course can be significantly further impaired in CD patients due to the pre-existing inflammation [5]. Therefore, it must be assumed that patients with CD also experience increased dysregulation of the pre-existing visceral hyperalgesia after surgical procedures. This assumption is supported by previous studies showing that CD patients had an increased need for opioids postoperatively compared to non-CD patients [6]. However, the significant limitation of these few studies that support the view that CD patients have increased patient experience in the perioperative setting, is that patients with malignant diseases have been used as control group instead of patients with non-IBD-related inflammation in the gut. Therefore, we hypothesized here that patients with chronic inflammation that is not caused by IBD compared to patients with IBD may indicate whether the need for higher analgesics in the perioperative setting is disease related. Based on this, the aim of this retrospective study is to analyze and compare postoperative pain and the need for analgesics in patients with Ileitis Crohn to patients suffering from diverticulitis after colorectal surgery. To provide an overview of the relationship between postoperative pain and analgesic requirements, the recently published analgesic score was applied [7].

## Materials and methods

### Study design

A single-centre retrospective study was carried out. All patients with CD or sigmoid diverticulitis (Non-IBD) who underwent open or minimal-invasive surgical care at the Department of Surgery at the University Hospital Würzburg between January 1, 2017, and December 31, 2021 were screened for possible data analysis. Inclusion criteria for the analysis were all patients aged 18 years and older who received elective open or minimal-invasive (laparoscopic) ileocoecal resection or sigmoid resection due to isolated ileitis terminalis Crohn and diverticulitis, retrospectively. Patients who did not have a complete data set or who required emergency surgery were excluded.

### Data acquisition

Clinical data were retrospectively obtained from the local database.

- Baseline patient characteristics: Age at operation, gender, Body Measure Index (BMI), preoperative depression and psychotherapy, preoperative (co-)analgesics use, laboratory values (hemoglobin, albumin), smoking, American Society of Anesthesiologists risk classification (ASA), Charlson Comorbidity Index (CCI), surgical approach and stoma were assessed.
- Preoperative immunosuppressive medication was also recorded and is listed in Appendix table 6. Antibody treatment was usually terminated 4 weeks before operation was performed. Medication with corticosteroids was also either terminated 2–4 weeks prior surgery or at least reduced to < 10 mg/day.
- Postoperative morbidity: Comprehensive Complication Index (CCI-Score) used to assess the overall morbidity of a patient after a surgical procedure on the basis of the Clavien-Dindo classification (CDC) [8], complications according to the Clavien-Dindo classification [9], other complications (postoperative ileus, wound infection, re-operation) and length of hospital stay of the patients were analysed.
- Postoperative pain and analgesics use: Peridural catheter (PDC) and duration, postoperative analgesics and duration (Paracetamol, metamizole, ibuprofen, opiate less and high potent, co-analgesics (included tricyclic antidepressants (amitriptyline, mirtazapine) and anticonvulsants (gabapentin, pregabalin)), psychotherapeutic consultation, pain level (using the numerical rating scale (NRS) from 0 (no pain) to 10 (worst pain imaginable)) at day 1/3/5/7/10 postoperative, analgesics score at day 3/5 and discharge.

### Analgesics score

To enable an objective comparison of the need for postoperative pain medication, we used the analgesics score which has been established previously for chronic postoperative groin pain after inguinal hernia surgery [7]. This score is based on the need for postoperative analgesics to achieve pain relief. In our study, we determined this score at three time points after surgery (day 3, day 5 and day of discharge). The analgesics taken at each time point were defined according to their efficacy using an ascending point scale based on the WHO analgesic ranking list and the dose taken. The analgesics score is the sum of all analgesic medications at a given time. In postoperative care, analgesic therapy was adjusted based on standard procedures until patients were pain-free at rest (NRS < 3). If a patient was not pain-free, analgesia was adjusted. This procedure was documented daily. To avoid bias due to analgesics and co-analgesics already taken preoperatively, these were subtracted from the total score according to their analgesic score point value.

In our patient population, the following co-analgesics were administered perioperatively or postoperatively based on individual clinical indications: Tricyclic antidepressants (Amitriptyline, Mirtazapine), Antiepileptic agents (Gabapentin, Pregabalin). These co-analgesics were prescribed in accordance with existing clinical guidelines and institutional protocols. Their use was tailored to the individual pain profile of each patient, with the aim of reducing opioid consumption and improving overall pain control. Their inclusion in the analgesics score reflects their recognized contribution to the multimodal pain regimen.

### Statistical analysis

Statistical analysis was performed using IBM SPSS 28.0 (IBM SPSS, Armonk, New York, USA). For the matched pair analysis, we carried out the statistical test of propensity score matching. Differences between groups were calculated using Welch’s test and Chi²-test as well as single factor variance and analysis of covariance or as repeated measure ANOVA. In the case of multiple T-tests, a post-hoc test was used by means of Bonferroni correction to detect individual comparisons between the groups without risking an alpha error. Significance was set at *p* < 0.05. Descriptive analyses included mean, minimum (min), and maximum (max) values given as range, percentage, and standard deviation.

To determine disease-related and disease-specific pain experience a comparison between patients with CD and Non-IBD, all 310 patients of both groups (CD or Non-IBD) were screened using the variables age, gender, and surgical procedure (minimally invasive or open). Pairs of one patient with CD and one Non-IBD patient with a deviation tolerance of 0 in all three variables (*p* = 1.000) were included. Matched-paired patients were then examined for all other variables as described above.

### Ethics approval

The study was conducted according to the guidelines of the Declaration of Helsinki and approved by the Institutional Ethics Committee of University of Würzburg, 97,080 Würzburg, Germany (Approval Number: 20240815 02).

## Results

### Characterization of the study population

**Fig. 1:**
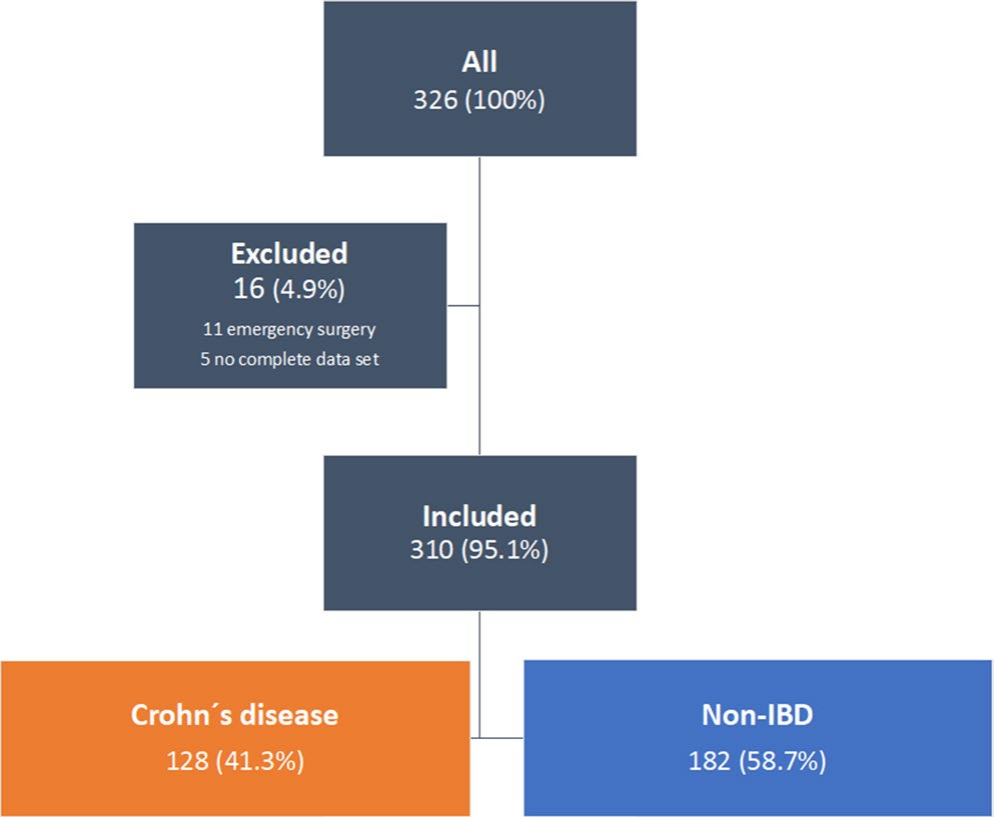
Study design

A total of 310 patients, 128 patients with CD and 182 patients with diverticulitis (non-IBD), were included and analysed. The classification of patients with sigmoid diverticulitis is shown in appendix 5. Overall, 16 (4.9%) patients were excluded from the data analysis: 3 patients died before hospital discharge (2 patients due to respiratory insufficiency, 1 patient due to severe sepsis), 11 patients underwent emergency surgery, and 2 patients had missing data (Fig. 1).

CD patients were significantly younger (37.5 years vs. 58.2 years; *p* < 0.001) and there were significantly more men in the CD cohort (54.7% vs. 42.3%; *p* = 0.032). CD patients had a significantly lower BMI (23.8 vs. 28.4; *p* < 0.001) and lower preoperative albumin level (4.4 vs. 4.2; *p* < 0.001) (Table 1). Additionally, Non-IBD patients were characterized by a significant higher ASA Score (2.37 vs. 1.95; *p* < 0.001) and a higher CCI (2.5 vs. 0.5; *p* < 0.001) [10] (Table 1).

**Table 1:** Baseline patient´s characteristics

	non-IBD (*n* = 182)	CD (*n* = 128)	*p*-Value
Sex			0.032
Male, n (%)	77 (42.3)	70 (54.7)	
Female, n (%)	105 (57.7)	58 (45.3)	
Age [years], mean (SD)	58.2 (12.9)	37.5 (15.2)	**0.001**
BMI [kg/m^2^], mean (SD)	28.4 (5.8)	23.8 (4.1)	**0.001**
Smoking, n (%)	40 (22)	33 (25.8)	0.437
Laboratory values [g/dl], mean (SD)			
Hemoglobin	13.4 (1.9)	13.0 (1.8)	0.089
Albumin	4.4 (0.5)	4.2 (0.5)	**0.001**
Depression, n (%)	12 (6.6)	8 (6.3)	0.904
Preoperative Medication, n (%)			
Non-opiate	30 (16.5)	8 (6.3)	**0.007**
Opiate (less potent)	12 (6.6)	1 (0.8)	**0.012**
Opiate (high potent)	1 (0.5)	1 (0.8)	0.802
Co-analgesics	12 (6.6)	9 (7)	0.880
Preoperative psychotherapy, n (%)	2 (1.1)	1 (0.8)	0.778
Surgical approach, n (%)			**0.002**
Minimal-invasive	140 (76.9)	78 (60.9)	
Open	42 (23.1)	50 (39.1)	
Stoma, n (%)	24 (13.2)	10 (7.8)	0.136

Interestingly, non-IBD patients reported a significantly higher need for non-opioids (16.5% vs. 6.3%; *p* = 0.007) and low-potent opioids (6.6% vs. 0.8%; *p* = 0.012) preoperatively compared to CD patients. Both groups showed an equally high rate of depression (6.6% versus 6.3%). Minimal-invasive surgical approach was significantly more frequently applied in non-IBD patients (76.9% vs. 60.9%; *p* = 0.002).

### Postoperative morbidity

No significant differences were detected regarding the CCI-Score (9.6 vs. 12.4; *p* = 0.119). However, there were significantly more postoperative complications CDD>3a in patients with CD compared to non-IBD (14.8% vs. 5.5%; *p* = 0.005). CD patients had a higher rate of anastomotic leakages (8.6% vs. 3.3%; *p* = 0.044), a higher re-operation rate (14.8% vs. 4.4%; *p* = 0.001) and more wound infections (19.5% vs. 11%; *p* = 0.036). However, both groups showed no significant difference in the length of hospital stay (9.3 days vs. 9.3 days; *p* = 0.901) (Table 2).

**Table 2:** Postoperative morbidity

	non-IBD(*n* = 182)	CD(*n* = 128)	*p*-Value
CCI-Score, mean (SD)	9.6 (14.9)	12.4 (16.6)	0.119
CDC >3a, n (%)	10 (5.5)	19 (14.8)	**0.005**
Anastomotic leakage, n (%)	6 (3.3)	11 (8.6)	**0.044**
Re-operation, n (%)	8 (4.4)	19 (14.8)	**0.001**
Postoperative ileus, n (%)	35 (19.2)	28 (21.9)	0.569
Wound infection, n (%)	20 (11)	25 (19.5)	**0.036**
Length of hospital stay [days], mean (SD)	9.26 (5.1)	9.34 (6.2)	0.901

### Postoperative pain and analgesics use

In both patient groups, rates of PDC use for pain management were comparable (15.9% vs. 14.8%, *p* = 0.794) but CD patients showed a significantly increased need for metamizole (89.1% vs. 73.1%; *p* < 0.001) and co-analgesics (18% vs. 4.4%; *p* < 0.001) postoperatively. 85.2% of the CD patients received a PDC for perioperative pain management and 84.1% of the non-IBD patients. While there was no significant increase in the need for low- and high-potent opioids in the CD cohort, the need for high-potent opioids for CD patients was significant increased compared to non-IBD patients (2.4 vs. 1.2 [days]; *p* = 0.038). (Table 3).

**Table 3:** Postoperative pain medication

	non-IBD (*n* = 182)	CD (*n* = 128)	*p*-Value
Peridural catheter			0.794
No, n (%)	29 (15.9)	19 (14.8)	
Yes, n (%)	153 (84.1)	109 (85.2)	
Duration [days], mean (SD)	3.6 (1.9)	3.9 (1.9)	0.244
Postoperative Medication			
Paracetamol, n (%)	108 (59.3)	88 (68.8)	0.091
Duration [days], mean (SD)	3.8 (5.0)	4.9 (6.3)	0.097
Metamizole, n (%)	133 (73.1)	114 (89.1)	**0.001**
Duration [days], mean (SD)	4.1 (4.7)	5.7 (6.4)	**0.012**
Ibuprofen, n (%)	12 (6.6)	4 (3.1)	0.174
Duration [days], mean (SD)	0.3 (1.0)	0.1 (0.7)	0.163
Opiate (less potent), n (%)	152 (83.5)	100 (78.1)	0.231
Duration [days], mean (SD)	3.5 (2.6)	3.3 (3.3)	0.547
Opiate (high potent), n (%)	38 (20.9)	35 (27.3)	0.187
Duration [days], mean (SD)	1.2 (3.1)	2.4 (6.7)	**0.038**
Co-analgesics, n (%)	8 (4.4)	23 (18)	**0.001**
Duration [days], mean (SD)	0.3 (1.4)	1.2 (3.7)	**0.002**
Psychotherapeutic consultation, n (%)	5 (2.7%)	2 (1.6%)	0.489

**Fig. 2:**
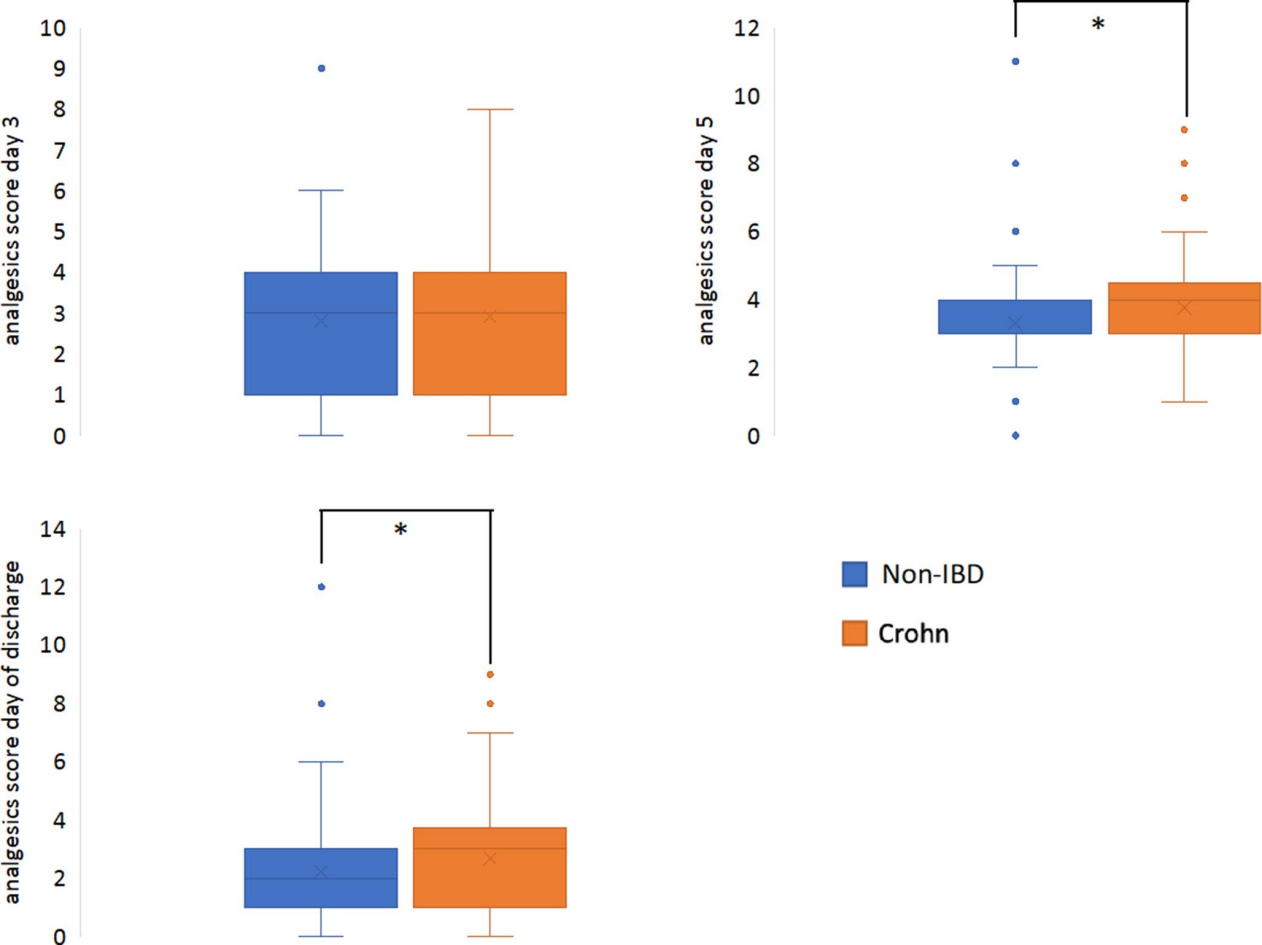
Postoperative analgesics scores, * *p* < 0.05

**Fig. 3:**
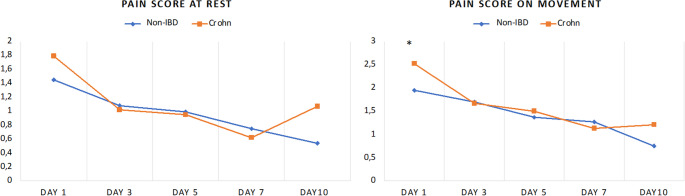
Postoperative subjective pain scores, * *p* < 0.05

While all patients were characterized by equal analgesic scores on day 3 after surgery, the analgesics score was significantly higher in patients with CD on day 5 postoperatively (3.75 vs. 3.29; *p* = 0.012) and on the day of discharge (2.70 vs. 2.24; *p* = 0.023) (Fig. 2). However, there was only one significant difference between the two groups regarding NRS scoring: CD patients reported a higher numerical value on the NRS for movement (2.52 vs. 1.95; *p* = 0.027) on day 1 after surgery. (Fig. 3).

### Matched pair analysis

After the match pairing based on the variables age, gender and surgical procedure (minimal-invasive or open approach), 27 patients with CD and 27 non-CD patients were identified and included. Patients with CD showed a significantly lower BMI (24.4 vs. 30.1; *p* < 0.001) and a significantly lower albumin value preoperatively (4.3 vs. 4.5; *p* = 0.032). Rates of depression and preoperative (co-)analgesic use were comparable (Table 4). Regarding postoperative complications, no significant differences were observed for CDD>3a (14.8% vs. 11.1%; *p* = 0.685), CCI-Score (10.7 vs. 14.4; *p* = 0.461) and length of hospital stay (11.2 days vs. 9.6 days; *p* = 0.482) (Table 4).

**Table 4:** Matched pair analysis

	non-IBD (*n*=27)	CD (*n*=27)	*p*-Value
*baseline patient´s characteristics*			
BMI [kg/m^2^], mean (SD)	30.1 (6.6)	24.4 (3.4)	**0.001**
Smoking, n (%)	8 (29.6)	9 (33.3)	0.770
Laboratory values [g/dl] mean (SD)			
Hemoglobin	13.5 (2.2)	13.5 (1.9)	0.990
Albumin	4.5 (0.5)	4.3 (0.4)	**0.032**
Depression, n (%)	4 (14.8)	2 (7.4)	0.334
Preoperative Medication, n (%)			
Non-opiate	3 (11.1)	0	0.075
Opiate (less potent)	2 (7.4)	1 (3.7)	0.500
Opiate (highpotent)	0	0	
Co-analgesics	4 (14.8)	3 (11.1)	0.500
Preoperative psychotherapy, n (%)	1 (3.7)	0	0.500
Stoma, n (%)	2 (7.4)	2 (7.4)	1.000
*postoperative morbidity*			
CCI-Score, mean (SD)	10.7 (15.4)	14.4 (20.7)	0.461
CDC >3a, n (%)	3 (11.1)	4 (14.8)	0.685
Anastomotic leakage, n (%)	1 (3.7)	4 (14.8)	0.159
Re-operation, n (%)	2 (7.5)	4 (14.8)	0.386
Postoperative ileus, n (%)	3 (11.1)	5 (18.5)	0.444
Wound infection, n (%)	4 (14.8)	5 (18.5)	0.715
Length of hospital stay [days], mean (SD)	9.6 (6.2)	11.2 (10.2)	0.482
*postoperative pain medication*			
*Peridural catheter*			0.125
No, n (%)	2 (7.4)	6 (22.2)	
Yes, n (%)	25 (92.6)	21 (77.8)	
Duration [days], mean (SD)	3.96 (1.6)	3.44 (2.1)	0.307
*Postoperative Medication*			
Paracetamol, n (%)	11 (40.7)	18 (66.7)	0.050
Duration [days], mean (SD)	3.5 (6.6)	6.2 (9.0)	0.206
Metamizole, n (%)	22 (81.5)	22 (81.5)	1.000
Duration [days], mean (SD)	4.1 (3.9)	6.9 (10.8)	0.209
Ibuprofen, n (%)	1 (3.7)	1 (3.7)	1.000
Duration [days], mean (SD)	0.2 (0.8)	0.1 (0.4)	0.657
Opiate (less potent), n (%)	24 (88.9)	22 (81.5)	0.444
Duration [days], mean (SD)	3.3 (2.2)	3.5 (4.5)	0.817
Opiate (high potent), n (%)	6 (22.2)	6 (22.2)	1.000
Duration [days], mean (SD)	1.1 (2.4)	4.0 (11.0)	0.188
Co-analgesics, n (%)	3 (11.1)	4 (14.8)	0.685
Duration [days], mean (SD)	0.7 (2.1)	0.8 (2.3)	0.902
Psychotherapeutic consultation, n (%)	0	1 (3.7)	0.500

**Fig. 4:**
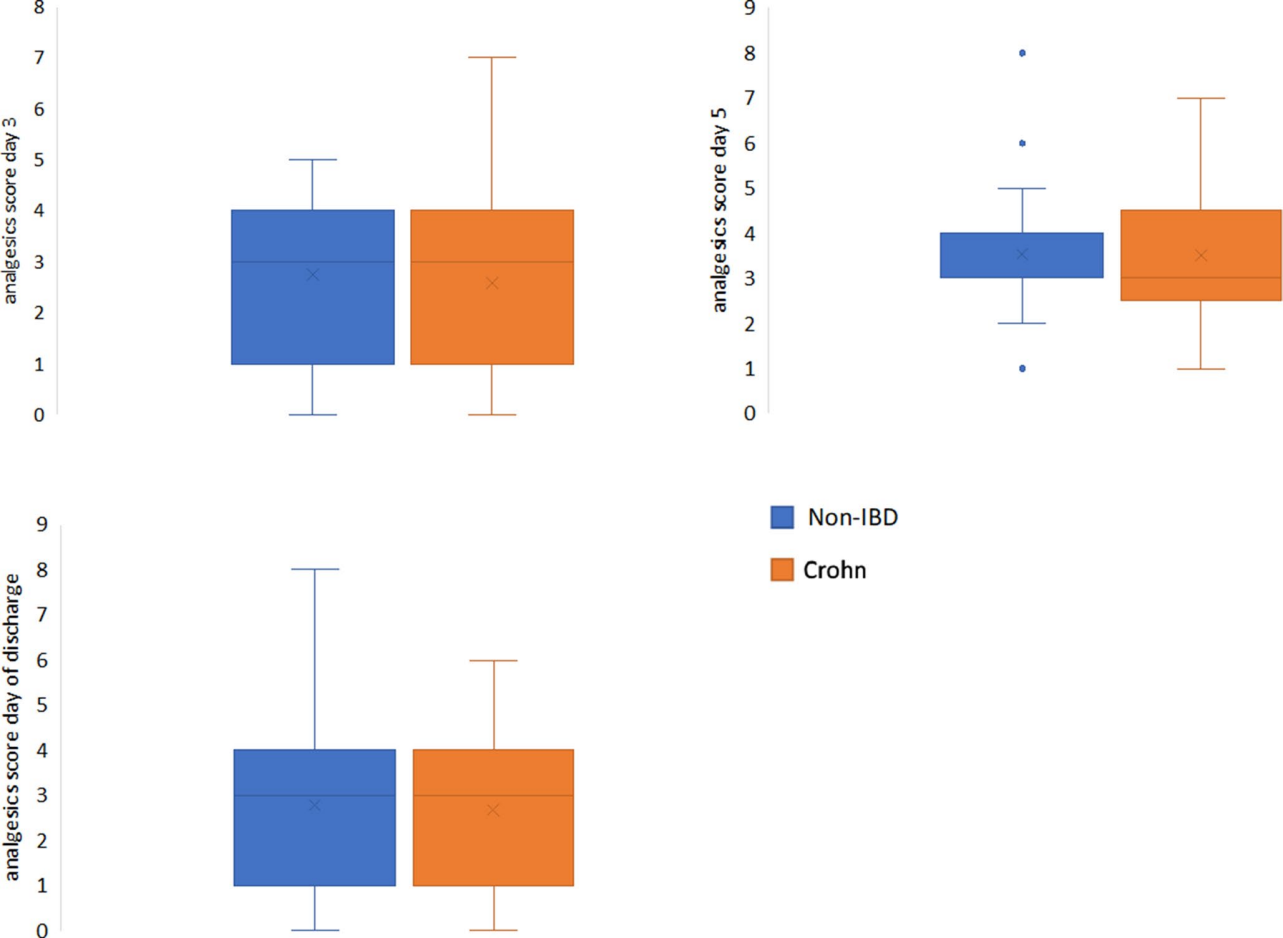
Matched pair analysis: Postoperative analgesics scores

**Fig. 5:**
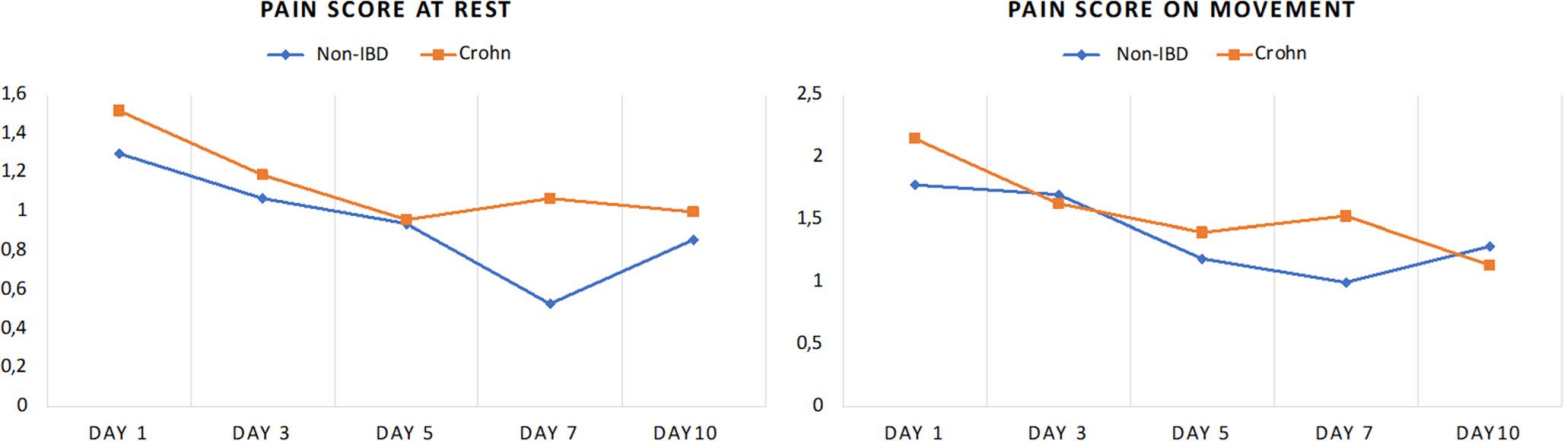
Matched pair analysis: Postoperative subjective pain scores

In both groups, the use of PDC was not different (92.6% vs. 77.8%; *p* = 0.125). There were no significant differences in postoperative analgesics use between both patient cohorts (Table 4). The analgesics score was similar for non-IBD patients and patients with CD at all three time points (3 days and 5 days after surgery and on the day of discharge) (Fig. 4). There were also no differences between the two groups in terms of pain ratings on the NRS for resting and movement (Fig. 5).

## Discussion

CD represents a non-curable disease with patients suffering from malnutrition as well as chronic psychological distress and pain. Since the pre-existing burden frequently aggravates postoperatively, adequate pain management is of great relevance for individual patients. To investigate the specific exposure of CD patients with ileitis terminalis, we analysed a matched cohort of non-IBD patients for postoperative pain and distress to identify relevant differences. Based on our findings and in contrast to previous studies using non-inflamed underlying diseases for comparison, our study may suggest that patients suffering from ileitis terminalis Crohn require a similar pain management than non-IBD patients. In difference to previous studies, we choose patients suffering from sigma diverticulitis as control group since this disease is also characterized by relapsing bowel inflammation accompanied with abdominal pain. To our mind this approach allows a more sufficient comparison to CD patients in contrast to cancer patients.

Although the non-IBD patient population shows a higher ASA-Classification and a higher CCI as well as a higher age and BMI, an increased complication rate was found in CD patients postoperatively for wound infections, anastomotic leakage, and an increased rate of re-operations. Those findings were mainly related to the fact that patients with CD are characterized by chronic inflammation, malnutrition, and immunosuppressive medication.

A previous study showed that the bacterial DNA translocation in CD patients caused by the chronic inflammatory process can explain an increased complication rate [11]. In addition low albumin levels associated with malnutrition have a negative impact on the postoperative outcome of CD patients as has been already shown in previous studies [11, 12]. In our study, CD patients also show a significantly lower preoperative albumin level than non-IBD patients. In addition, steroid therapies or immunosuppressive medication are significantly more often used for treatment in CD patients increasing the perioperative risk of complications [13]. However, there was no prolonged hospital stay for CD patients since the failure to rescue rate was low and many postoperative complications such as wound infection could be treated in an outpatient clinic.

To analyse postoperative sensation of pain we used the well-known NRS but also the recently established analgesics score which is already established in patients with pain after inguinal hernia repair [7]. The advantage of this novel score is the capacity to correlate individual patients´ pain and the need of analgesia (pain reliver). Thus, analgesics score provides information on pain levels and the necessary analgesic therapy and finally avoids an evaluation that is solely based on patient reporting (NRS). Furthermore, we suggest that this score is not restricted to patients with hernia but appears to be applicable for other surgeries to assess additional dimensions of pain experience. This could complement the NRS in the future.

Primary analysis for postoperative analgesic use including all patients reveals that CD patients have a significantly higher and longer need of metamizole, high potent opioids, and co-analgesics compared to non-IBD patients. Furthermore, analgesics scores are significantly higher in CD patients on day 5 postoperatively and on the day of discharge indicating that CD patients need an intensified analgesic regimen due to increased pain postoperatively. These observations are in line with previously published studies [6, 14]. However, since our cohort is characterized by significant differences in age, sex and the surgical approach (minimal-invasive or open approach) between the two groups (CD versus non-IBD), we performed a matched-pair analysis to exclude potential factors influencing postoperative pain sensation and pain medication. Interestingly, the matched-pair analysis does not show any difference regarding postoperative pain, analgesics use and analgesics score comparing CD and non-IBD patients. Thus, patients suffering from limited inflammatory conditions independently of aetiology may require similar postoperative pain management after colorectal surgery. Larger cohorts of patients and prospective evaluation of the postoperative pain management in CD patients will be required to substantiate this conclusion.

### Strength and limitations

The main limitations of our study are its retrospective nature and the single-center design. Based on the character of our study there is always a risk of bias and confounding. However, the study includes one of the largest patient cohorts on this issue comparing similar disease phenotypes. This allows for the first time a sufficient representation of real-life conditions and, therefore, provides reliable results. The matched pair analysis enabled a rudimentary group comparison with an appropriate group size. However, further confounders such as BMI, ASA, preoperative analgesic use, preoperative immunosuppressive therapy (e.g. antibody therapy, steroids) could be not considered in the matched pair analysis since size of matched groups would be too small to draw any conclusions.

Furthermore, our study did not investigate if patients suffering from pre-existing visceral hypersensitivity. However, we would have expected a higher need of preoperative and postoperative pain medication in CD patients if they would have been suffering from visceral hypersensitivity, and thus, from altered pain sensation and processing. Also, biochemical aspects such as concentration of pro-inflammatory cytokines or receptors that are well known to facilitate pain (e.g. bradykinin and its receptors) could not been evaluated in our study due to the retrospective character [15]. Here, further prospective translational studies are required to adequately answer this research topic.

Since previous studies evaluating postoperative pain in CD patients used patients suffering from cancer as a control group, we decided to include only patients with diverticulitis since these patients are also characterized by a relapsing inflammation of the bowel. Here of course, it must be acknowledged that surgical magnitude, extraction site length and specimen removal technique can differ between ileocoecal and sigmoid resection, and thus, influence postoperative pain sensation. However, in our opinion it would be very challenging to exclude these factors due to the nature of diseases with a high variation of inflammation, and patient specific characteristics such as BMI, visceral obesity and previous operations.

## Conclusion

Adequate pain management is of great relevance for patients suffering from a chronic disease burden such as CD. Based on our results, postoperative analgesic regimen need to consider the individual patient’s pain score, pain experience and sensation irrespective of the underlying disease leading to colorectal surgery. In detail, patients with limited ileitis terminalis Crohn may not be different to patients suffering from diverticulitis, even if the complication rate tends to be increased in CD patients. Further prospective studies are mandatory to confirm our observations and to evaluate the individual pain sensation of patients.

## Figures and Tables

**Fig. 6:**
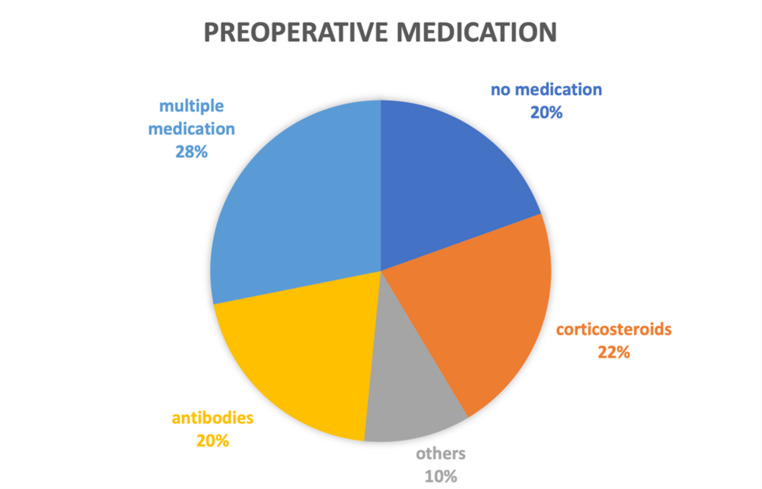
Perioperative Crohn-specific medication

## Data Availability

The data that support the findings of this study are available on request from the corresponding author. The data are not publicly available due to privacy or ethical restrictions.

## Appendix

**Table 5:** Classification of sigmoid diverticulitis in patients with sigmoid diverticulitis

CDD1: acute uncomplicated, *n* (%)	0
CDD 2a/b: acute complicated with abscess, n (%)	68 (37.4)
CDD 2c: with free perforation, n (%)	2 (1.1)
CDD 3: chronic diverticular disease, n (%)	112 (61.5)

**Table 6:** Perioperative Crohn-specific medication

no medication, *n* (%)	25 (20)
corticosteroids, n (%)	28 (22)
antibodies, n (%)	13 (10)
others, n (%)	26 (20)
multiple medication, n (%)	36 (28)

## References

[1] Mayer EA, Gebhart GF (1994) Basic and clinical aspects of visceral hyperalgesia. Gastroenterology 107(1):271–293 8020671 10.1016/0016-5085(94)90086-8

[2] Faes S, Hahnloser D (2018) Chirurgische Behandlung der chronisch entzündlichen Darmerkrankungen. Therapeutische Umschau 75(5):302–314 10.1024/0040-5930/a001001 30700243

[3] Sido B et al (2004) Inflammatory response after abdominal surgery. Best Pract Res Clin Anaesthesiol 18(3):439–454 15212338 10.1016/j.bpa.2003.12.006

[4] Lahiri R et al (2016) Systemic inflammatory response syndrome after major abdominal surgery predicted by early upregulation of TLR4 and TLR5*.* Ann Surg 263(5):1028–1037 10.1097/SLA.0000000000001248 26020106

[5] de Buck A et al (2015) Postoperative inflammatory response in Crohn’s patients: a comparative study. J Crohn’s Colitis 9(12):1127–1131 26351389 10.1093/ecco-jcc/jjv161

[6] Guidat A et al (2003) Inflammation increases sufentanil requirements during surgery for inflammatory bowel diseases. Eur J Anaesthesiol 20(12):957–962 14690097 10.1017/s0265021503001546

[7] Widder A et al (2023) Postoperative Analgesics Score as a Predictor of Chronic Postoperative Inguinal Pain After Inguinal Hernia Repair: Lessons Learned From a Retrospective Analysis. World J Surg 47(10):2436–2443 37248322 10.1007/s00268-023-07074-6 PMC10474177

[8] Slankamenac K et al (2013) The comprehensive complication index: a novel continuous scale to measure surgical morbidity. Ann Surg 258(1):1–7 23728278 10.1097/SLA.0b013e318296c732

[9] Dindo D (2014) The Clavien–Dindo classification of surgical complications*.* Treatment of postoperative complications after digestive surgery, pp. 13–17

[10] Charlson ME et al (1987) A new method of classifying prognostic comorbidity in longitudinal studies: development and validation. J chronic Dis 40(5):373–383 3558716 10.1016/0021-9681(87)90171-8

[11] Li Y et al (2015) The impact of bacterial DNA translocation on early postoperative outcomes in Crohn’s patients undergoing abdominal surgery. J Crohn’s Colitis 9(3):259–265 25555386 10.1093/ecco-jcc/jju029

[12] Nguyen GC et al (2019) Hypoalbuminaemia and postoperative outcomes in inflammatory bowel disease: the NSQIP surgical cohort. J Crohn’s Colitis 13(11):1433–1438 31253985 10.1093/ecco-jcc/jjz083 PMC6821313

[13] Bakes D, Kiran RP (2022) Overview of Common Complications in Inflammatory Bowel Disease Surgery. Gastrointest Endoscopy Clin 32(4):761–776 10.1016/j.giec.2022.05.011 36202515

[14] Huehne K et al (2009) High post surgical opioid requirements in Crohn’s disease are not due to a general change in pain sensitivity. Eur J Pain 13(10):1036–1042 19167252 10.1016/j.ejpain.2008.12.004

[15] Stadnicki A, Pastucha E, Nowaczyk G, Mazurek U, Plewka D, Machnik G, Wilczok T, Colman RW (2005) Immunolocalization and expression of kinin B1R and B2R receptors in human inflammatory bowel disease. Am J Physiol Gastrointest Liver Physiol 289(2):G361–G366 15805101 10.1152/ajpgi.00369.2004

